# Author Correction: Feasibility of whole genome and transcriptome profiling in pediatric and young adult cancers

**DOI:** 10.1038/s41467-025-63695-6

**Published:** 2025-09-03

**Authors:** N. Shukla, M. F. Levine, G. Gundem, D. Domenico, B. Spitzer, N. Bouvier, J. E. Arango-Ossa, D. Glodzik, J. S. Medina-Martínez, U. Bhanot, J. Gutiérrez-Abril, Y. Zhou, E. Fiala, E. Stockfisch, S. Li, M. I. Rodriguez-Sanchez, T. O’Donohue, C. Cobbs, M. H. A. Roehrl, J. Benhamida, F. Iglesias Cardenas, M. Ortiz, M. Kinnaman, S. Roberts, M. Ladanyi, S. Modak, S. Farouk-Sait, E. Slotkin, M. A. Karajannis, F. Dela Cruz, J. Glade Bender, A. Zehir, A. Viale, M. F. Walsh, A. L. Kung, E. Papaemmanuil

**Affiliations:** 1 Department of Pediatrics, Memorial Sloan Kettering Cancer Center, New York, NY USA; 2 Department of Epidemiology & Biostatistics, Memorial Sloan Kettering Cancer Center, New York, NY USA; 3 Department of Pathology, Memorial Sloan Kettering Cancer Center, New York, NY USA; 4 Precision Pathology Biobanking Center, Memorial Sloan Kettering Cancer Center, New York, NY USA; 5 Department of Medicine, Memorial Sloan Kettering Cancer Center, New York, NY USA; 6 Integrated Genomics Operation Core, Center for Molecular Oncology, Memorial Sloan Kettering Cancer Center, New York, NY USA; 7 Human Oncology and Pathogenesis Program, Memorial Sloan Kettering Cancer Center, New York, NY USA

**Keywords:** Cancer genomics, Paediatric cancer, Computational biology and bioinformatics

Correction to: *Nature Communications* 10.1038/s41467-022-30233-7, published online 18 May 2022

In the version of this article originally published, there were labeling mistakes in the samples and patient identification numbers presented in the article. Text in the article, Supplementary Information and Supplementary Data have now been amended in the HTML and PDF versions of the article.

