# Supplementary material for: Feasibility of whole genome and transcriptome profiling in pediatric and young adult cancers
Source: Nat Commun. 2022 May 18;13:2485. doi: 10.1038/s41467-022-30233-7 (PMC9117241; doi:10.1038/s41467-022-30233-7)
Supplement: Supplementary file 1 — Supplementary Information [file 41467_2022_30233_MOESM1_ESM.pdf]

S.Figure 1

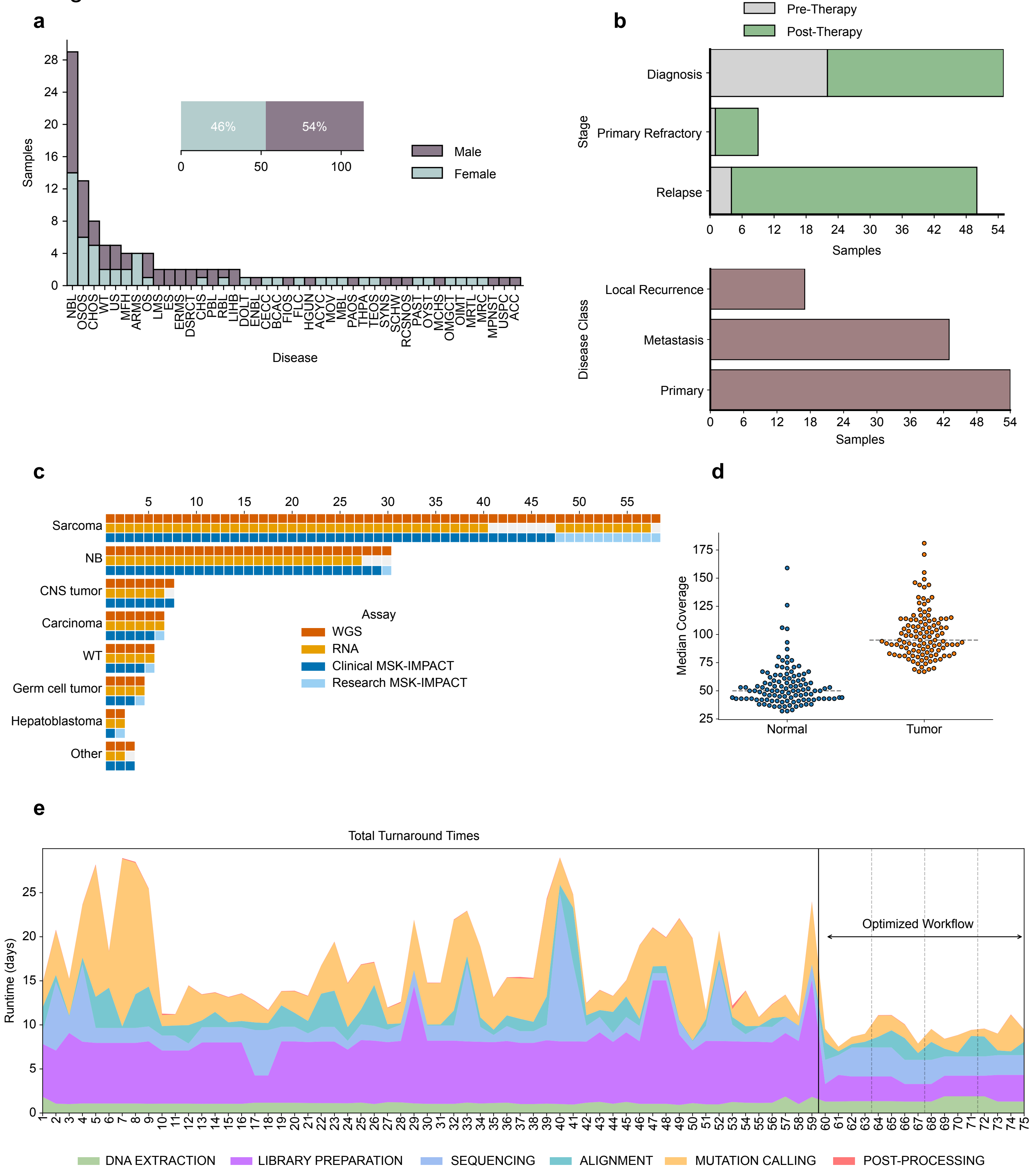
a
Samples
Disease
46%
54%
Male
Female
b
Therapy
Pre-Therapy
Post-Therapy
Stage
Diagnosis
Primary Refractory
Relapse
Samples
Disease Class
Local Recurrence
Metastasis
Primary
c
Sarcoma
NB
CNS tumor
Carcinoma
WT
Germ cell tumor
Hepatoblastoma
Other
Assay
WGS
RNA
Clinical MSK-IMPACT
Research MSK-IMPACT
d
Median Coverage
Normal
Tumor
e
Total Turnaround Times
Runtime (days)
Optimized Workflow
DNA EXTRACTION
LIBRARY PREPARATION
SEQUENCING
ALIGNMENT
MUTATION CALLING
POST-PROCESSING

Supplementary Figure 1: Study cohort overview a) Bar chart displaying distribution of patients across main oncotree disease code and color-coded by gender (ACC: Adrenocortical Carcinoma, ACYC: Adenoid Cystic Carcinoma, ARMS: Alveolar Rhabdomyosarcoma, BCAC: Basal cell adenocarcinoma, CECC: Cervical Clear Cell Carcinoma, CHOS: Chondroblastic Osteosarcoma, CHS: Chondrosarcoma, DOLT: Disseminated Oligodendroglial like Leptomeningeal Tumor, DSRCT: Desmoplastic Small-Round-Cell Tumor, ENBL: Esthesioneuroblastoma, ERMS: Embryonal Rhabdomyosarcoma, ES: Ewing Sarcoma, FIOS: Fibroblastic Osteosarcoma, FLC: Fibrolamellar Carcinoma, HGUN: Undifferentiated Neoplasm, LIHB: Hepatoblastoma, LMS: Leiomyosarcoma, MBL: Medulloblastoma, MCHS: Mesenchymal Chondrosarcoma, MFH: Undifferentiated Pleomorphic Sarcoma/Malignant Fibrous Histiocytoma/High-Grade Spindle Cell Sarcoma, MOV: Mucinous Ovarian Cancer, MPNST: Malignant Peripheral Nerve Sheath Tumor, MRC: Renal Medullary Carcinoma, MRTL: Malignant Rhabdoid Tumor of the Liver, NBL: Neuroblastoma, OIMT: Immature Teratoma, OMGCT: Mixed Germ Cell Tumor, OS: Osteosarcoma, OSOS: Osteoblastic Osteosarcoma, OYST: Yolk Sac Tumor, PAOS: Parosteal Osteosarcoma, PAST: Pilocytic Astrocytoma, PBL: Pineoblastoma, RBL: Retinoblastoma, RCSNOS: Round Cell Sarcoma, NOS, SCHW: Schwannoma, SYNS: Synovial Sarcoma, TEOS: Telangiectatic Osteosarcoma, THPA: Papillary Thyroid Cancer, US: Undifferentiated Sarcoma, USPC: Undifferentiated Sarcoma of the Peritoneal Cavity, WT: Wilms Tumor). b) Bar charts giving details about treatment status by disease stage (top) and tumor location (bottom) for n=114 tumors in the cohort. c) Tile plot summarizing molecular profiling assays run on each of the patient samples. Clinical MSK-IMPACT is used when available, otherwise Research MSK-IMPACT was used. d) Swarm style plot showing median coverage assessed by Mosdepth for the WGS sequencing data derived from tumor and normal samples in study (n=114). The dotted line indicates the median across samples. e) Sediment plot showing breakdown of different runtime processes contributing to total turnaround time for the set of 59 tumors with available data during optimization and the 16 tumors processed with the final optimized workflow. Source data for panels a-c are provided in Supplementary Data 1. Source data for panels d and e are provided at the data repository.

S.Figure 2

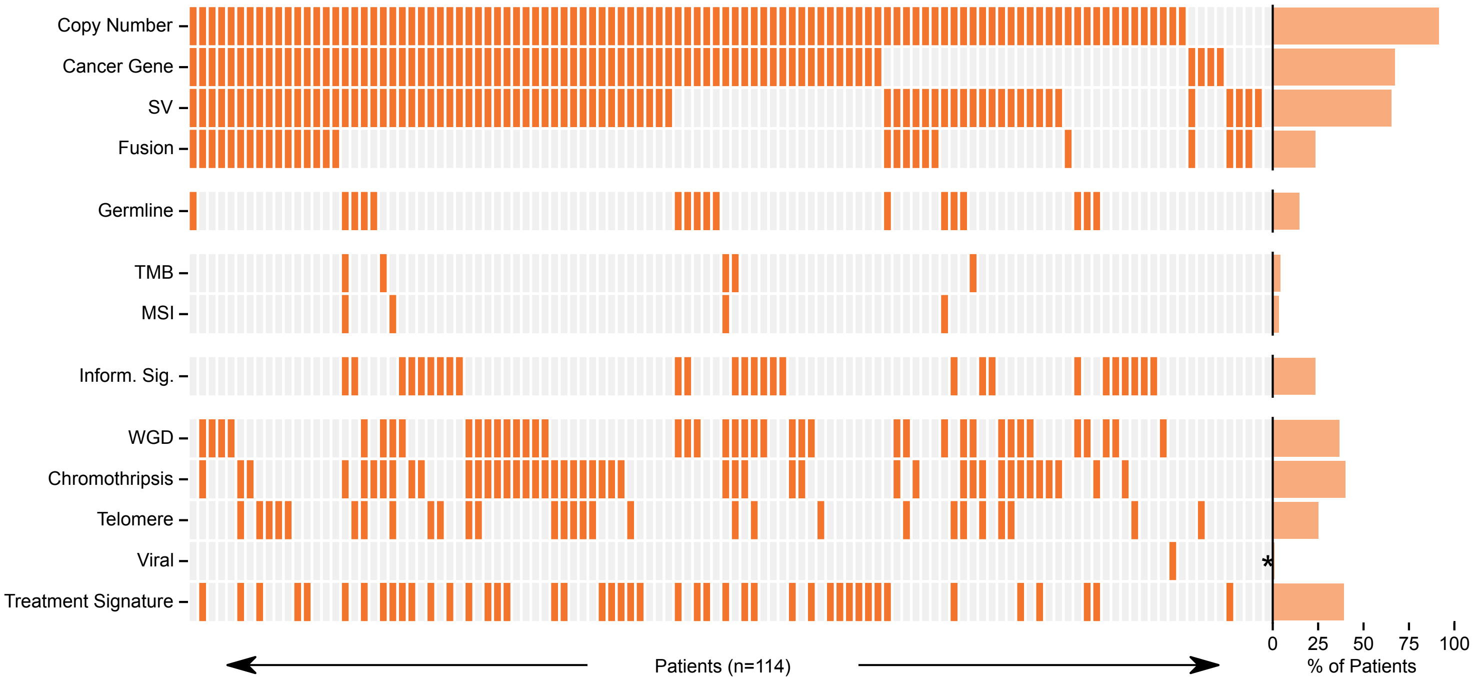
Copy Number
Cancer Gene
SV
Fusion
Germline
TMB
MSI
Inform. Sig.
WGD
Chromothripsis
Telomere
Viral
Treatment Signature
*
0
25
50
75
100
% of Patients
Patients (n=114)

Supplementary Figure 2: cWGTS biomarker overview a) Heatmap representing the presence of genomic markers by cWGTS, with columns as samples and rows as markers, to include: 1. Somatic copy number aberrations (CNA). 2. Somatic SNVs/indels affecting cancer genes (Cancer Gene). 3. Somatic structural variants targeting cancer genes (SV). 4. Somatic oncogenic gene fusions (Fusion). 5. Clinically relevant SNVs and indels in established germline predisposition genes (Germline). 6. Coding tumor mutation burden (TMB). 7. Microsatellite instability (MSI). 8. Informative signatures (e.g., mutation signatures associated with mutations in DNA repair genes) (Inform. Sig.). 9. Whole genome duplication (WGD). 10. Chromothripsis. 11. Aberrant telomere length between germline and tumor samples (Telomere). 12. Evidence of viral integration sequences in RNAseq (Viral). 13. Treatment-related signatures (e.g., mutation signatures associated with platinum or temozolomide exposure in tumor) (Treat. Sig.) Bar plot on the right shows the proportion of patients whose tumor harbors each genomic marker. The single sample in our cohort for which an oncogenic driver was not found, was an Epstein-Barr virus (EBV) driven leiomyosarcoma tumor for which RNA-seq was not available for assessment of EBV-derived sequences. Source data for this figure are provided in Supplementary Data 3.

S.Figure 3

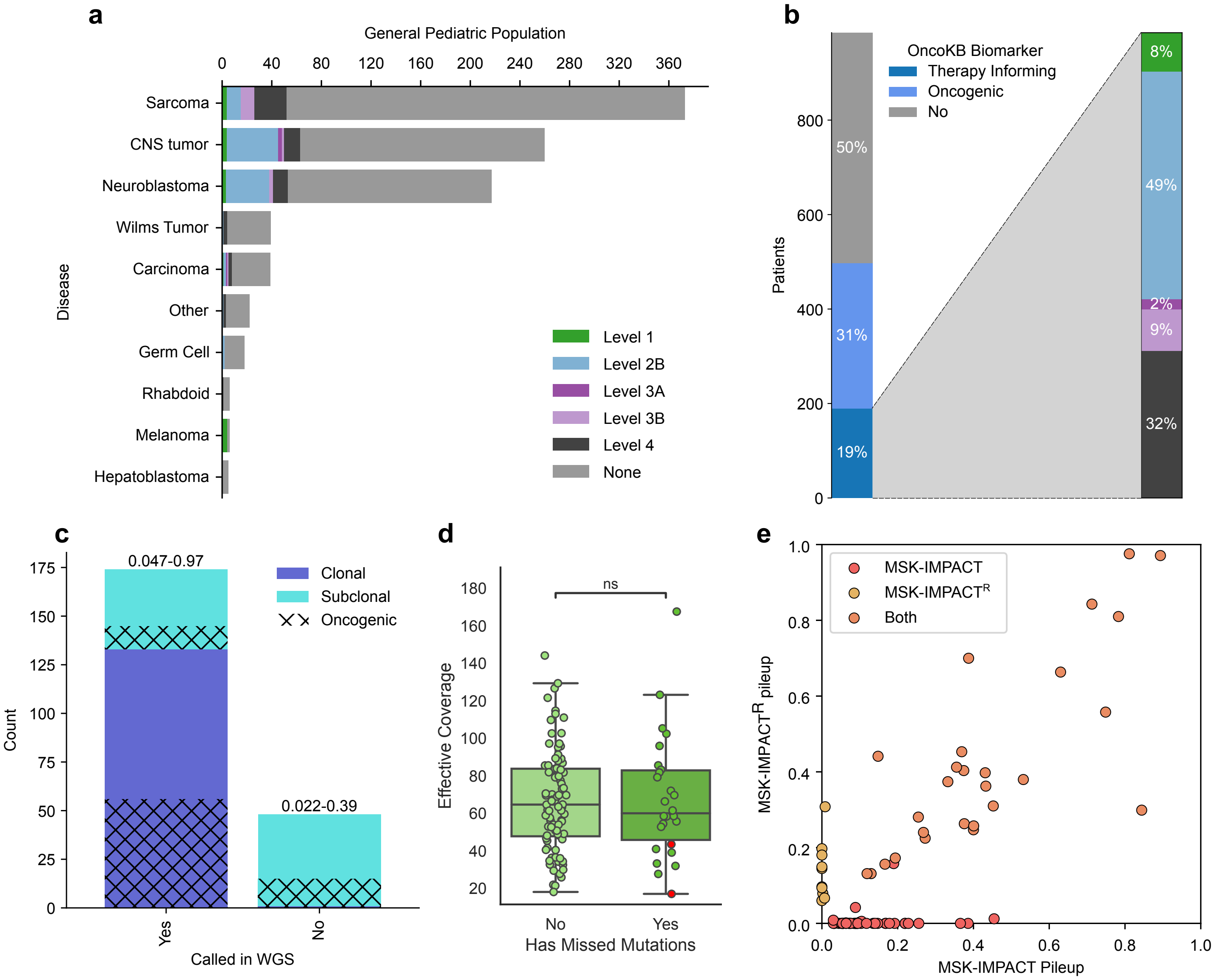

a
General Pediatric Population
0
40
80
120
160
200
240
280
320
360
Disease
Sarcoma
CNS tumor
Neuroblastoma
Wilms Tumor
Carcinoma
Other
Germ Cell
Rhabdoid
Melanoma
Hepatoblastoma
Level 1
Level 2B
Level 3A
Level 3B
Level 4
None
b
OncoKB Biomarker
Therapy Informing
Oncogenic
No
Patients
800
600
400
200
0
50%
31%
19%
8%
49%
2%
9%
32%
c
Count
175
150
125
100
75
50
25
0
0.047-0.97
0.022-0.39
Clonal
Subclonal
Oncogenic
Yes
No
Called in WGS
d
Effective Coverage
180
160
140
120
100
80
60
40
20
ns
No
Yes
Has Missed Mutations
e
MSK-IMPACT
MSK-IMPACT^R
Both
MSK-IMPACT^R pileup
1.0
0.8
0.6
0.4
0.2
0.0
MSK-IMPACT Pileup

Supplementary Figure 3: Analytical validity of cWGTS extended a) Bar plot displaying proportion of patients with OncoKb findings broken down by disease category for the extended pediatric and young adult patient cohort (n=985, median age 10.75, range: 0-39.3) at MSKCC. b) Bar plot displaying overall proportion of patients with OncoKb findings in the extended cohort (n=985). c) Bar plot displaying clonality status (clonal or subclonal), and oncogenic relevance of mutations reported by MSK-IMPACT broken down by whether the mutation was also called by WGS. d) Boxplot displaying WGS effective coverage (purity x coverage) for samples with small mutation (n=87) in study cohort broken down by whether all MSK-IMPACT mutations were called or not. Center line indicates the median across all samples and whiskers extend to within +/- 1.5 x the interquartile range. NS, not significant. e) Scatter plot showing VAF for mutations called by clinical and/or resequenced MSK-IMPACT. Source data for panel c are provided in Supplementary Data 4. Source data for d and e are provided at the data repository.

S.Figure 4

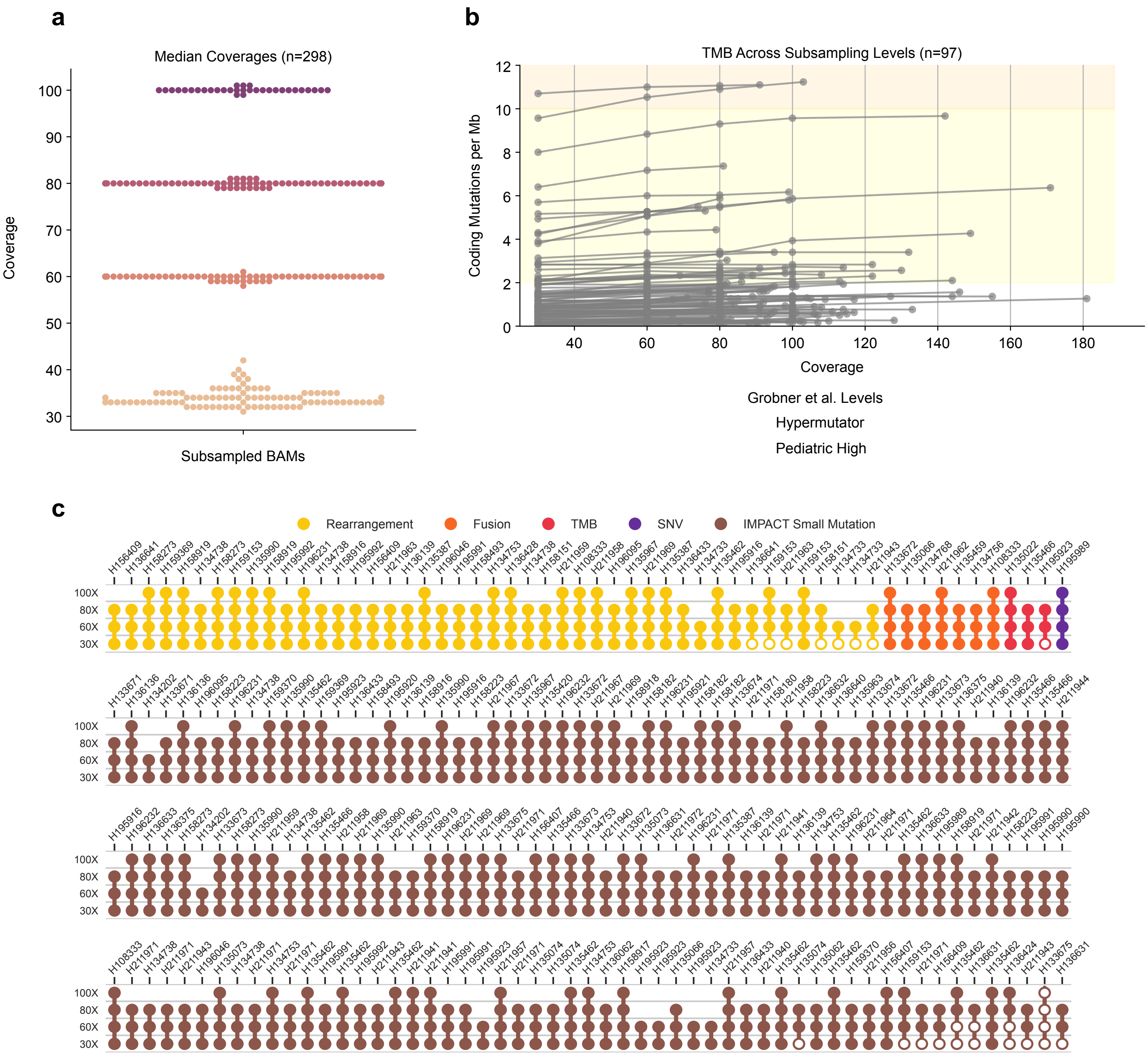
a
Median Coverages (n=298)
Coverage
Subsampled BAMs
b
TMB Across Subsampling Levels (n=97)
Coding Mutations per Mb
Coverage
Grobner et al. Levels
Hypermutator
Pediatric High
c
Rearrangement
Fusion
TMB
SNV
IMPACT Small Mutation
100X
80X
60X
30X

Supplementary Figure 4: Subsampling benchmarking analysis a) Distribution of median coverages for BAM files at each sub-sampling level. (32 to 100x, 72 to 80x, 97 to 60x and below) b) TMB across coding mutations per sample at each subsampled level. c) Recapitulated, clinically relevant findings at each subsampling level (n=220) color coded by mutation class and labeled with Individual ID. Rearrangements and Fusions are ordered by decreasing read support and MSK-IMPACT mutations are ordered by decreasing Variant Allele Fraction. Source data for panel a are provided in Supplementary Data 7. Source data for b-c are provided at the data repository.

S.Figure 5

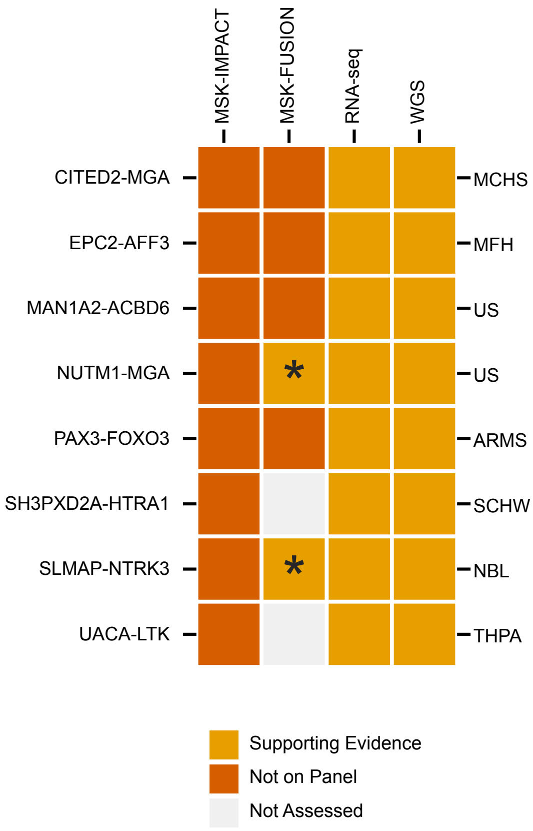
MSK-IMPACT
MSK-FUSION
RNA-seq
WGS
CITED2-MGA
EPC2-AFF3
MAN1A2-ACBD6
NUTM1-MGA
PAX3-FOXO3
SH3PXD2A-HTRA1
SLMAP-NTRK3
UACA-LTK
MCHS
MFH
US
US
ARMS
SCHW
NBL
THPA
*
*
Supporting Evidence
Not on Panel
Not Assessed

Supplementary Figure 5: Fusions identified by cWGTS. Heatmap demonstrating 8 additional fusions detected by cWGTS. Of these, 2 (*NUTM1-MGA* and *SLMAP-NTRK3*) were subsequently validated by MSK-Fusion. ARMS: Alveolar Rhabdomyosarcoma, NBL: Neuroblastoma, MCHS: Mesenchymal Chondrosarcoma, MFH: Malignant Fibrous Histiocytoma, SCHW: Schwannoma, THPA: Papillary Thyroid Cancer, US: Undifferentiated Sarcoma. Source data for this figure are provided in Supplementary Data 6.

S.Figure 6

a

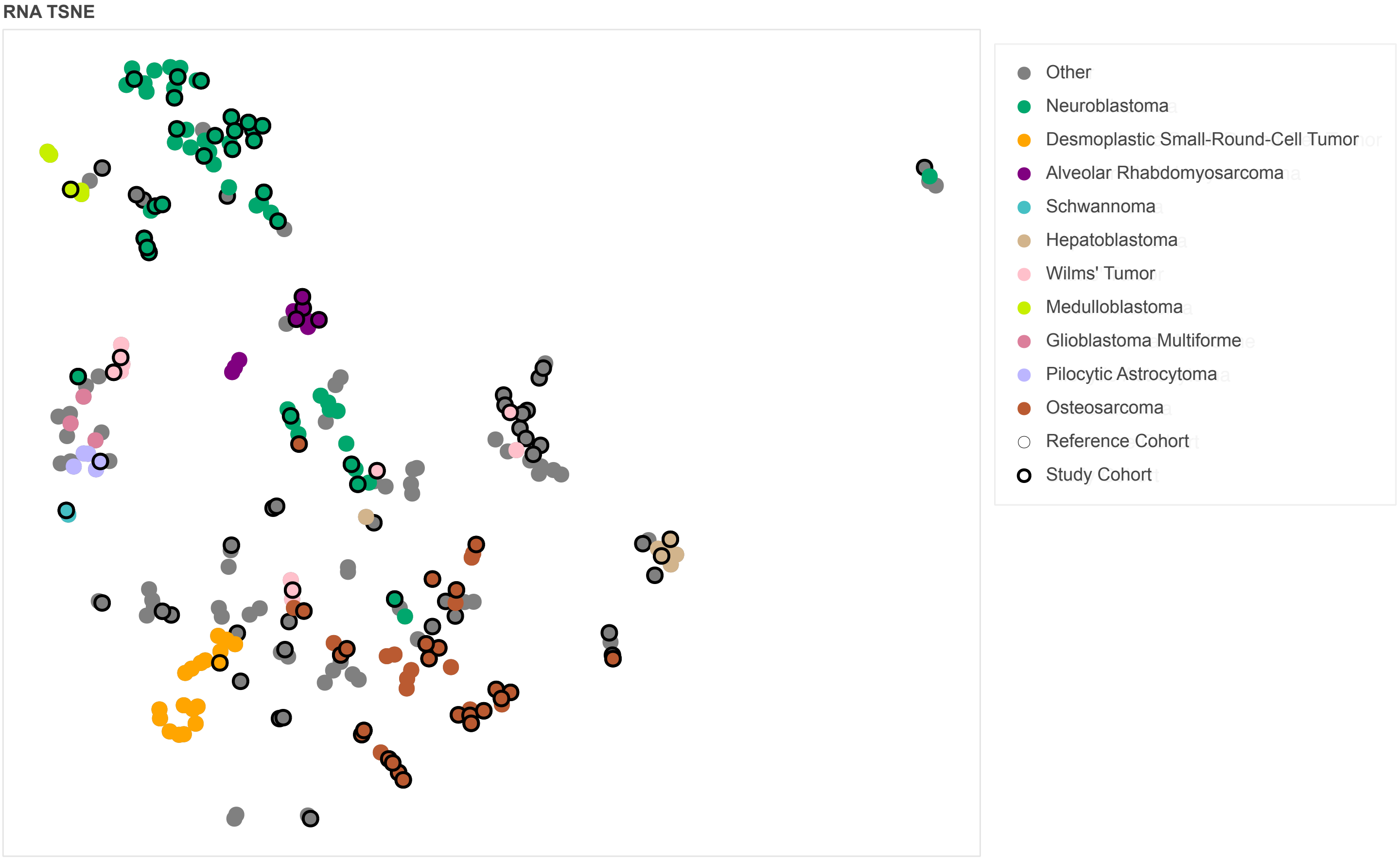

RNA TSNE
Other
Neuroblastoma
Desmoplastic Small-Round-Cell Tumor
Alveolar Rhabdomyosarcoma
Schwannoma
Hepatoblastoma
Wilms' Tumor
Medulloblastoma
Glioblastoma Multiforme
Pilocytic Astrocytoma
Osteosarcoma
Reference Cohort
Study Cohort

b

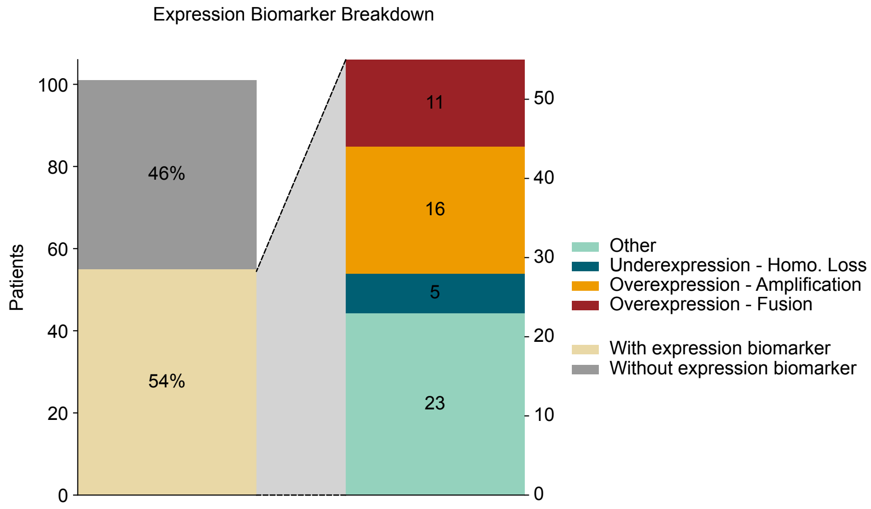

Expression Biomarker Breakdown
Patients
100
80
60
40
20
0
46%
54%
11
16
5
23
50
40
30
20
10
0
Other
Underexpression - Homo. Loss
Overexpression - Amplification
Overexpression - Fusion
With expression biomarker
Without expression biomarker

Supplementary Figure 6: RNA Clustering. a) t-distributed Stochastic Neighbor Embedding (TSNE) map of RNA expression data from study (n=101) and extended in-house reference cohort (n=155) colored by selected disease groups. b) Left bar plot shows breakdown of patients with >= 1 expression biomarker supported by an SV event while right bar plot shows the different categories of associated SV events (Homo. Loss = Homozygous Loss). Raw data for panel a can be accessed at the dbGAP study. Source data for panel b are provided in Supplementary Data 3.

S.Figure 7

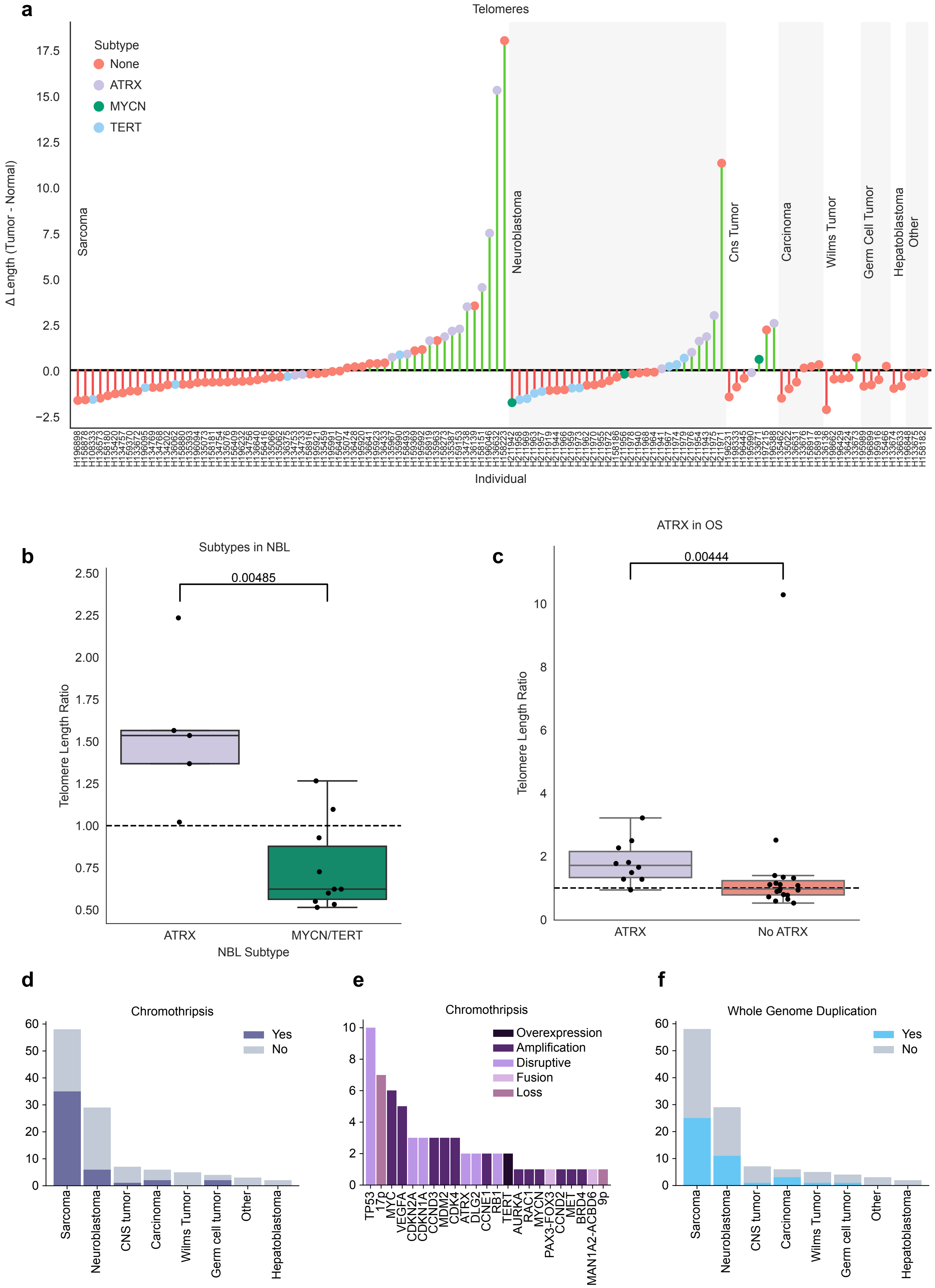
a
Telomeres
Subtype
None
ATRX
MYCN
TERT
Δ Length (Tumor - Normal)
Sarcoma
Neuroblastoma
Cns Tumor
Carcinoma
Wilms Tumor
Germ Cell Tumor
Hepatoblastoma
Other
Individual
b
Subtypes in NBL
0.00485
Telomere Length Ratio
ATRX
MYCN/TERT
NBL Subtype
c
ATRX in OS
0.00444
Telomere Length Ratio
ATRX
No ATRX
d
Chromothripsis
Yes
No
Sarcoma
Neuroblastoma
CNS tumor
Carcinoma
Wilms Tumor
Germ cell tumor
Other
Hepatoblastoma
e
Chromothripsis
Overexpression
Amplification
Disruptive
Fusion
Loss
TP53
17p
MYC
VEGFA
CDKN2A
CDKN1A
CCND3
MDM2
CDK4
ATRX
DLG2
CCNE1
RB1
TERT
AURKA
RAC1
MYCN
PAX3-FOXO3
CCND2
MET
BRD4
MAN1A2-ACBD6
9p
f
Whole Genome Duplication
Yes
No
Sarcoma
Neuroblastoma
CNS tumor
Carcinoma
Wilms Tumor
Germ cell tumor
Other
Hepatoblastoma

Supplementary Figure 7: Telomere length analysis a) Difference in telomere length between the tumor and matching normal sample as assessed by WGS. Increase in telomere length is shown with green and decrease with red. The individuals are colored by oncogenic events (ATRX: *ATRX* mutation or SV, MYCN: *MYCN* amplification, TERT: *TERT* promoter mutation or SV). b) Boxplots show telomere length ratio (tumor divided by normal) in neuroblastoma tumors with different oncogenic events. c) Telomere length ratio for Osteosarcoma patients with and without *ATRX* mutations (n=29). For both b and c, the center line indicates the median while whiskers extend to the data within +/- 1.5x interquartile range (n=15) (Wilcoxon rank sum test). d) Distribution of chromothripsis across disease groups. e) Genes affected by chromothripsis events colored by effect (Amplification: copy number gain in DNA, Disruptive: truncating structural variant, Fusion: found in RNA, Loss: copy number loss in DNA). f) Distribution of whole genome duplication across disease groups. Source data for a-c are provided at the data repository. Source data for panel d-f are provided in Supplementary Data 3.

S.Figure 8

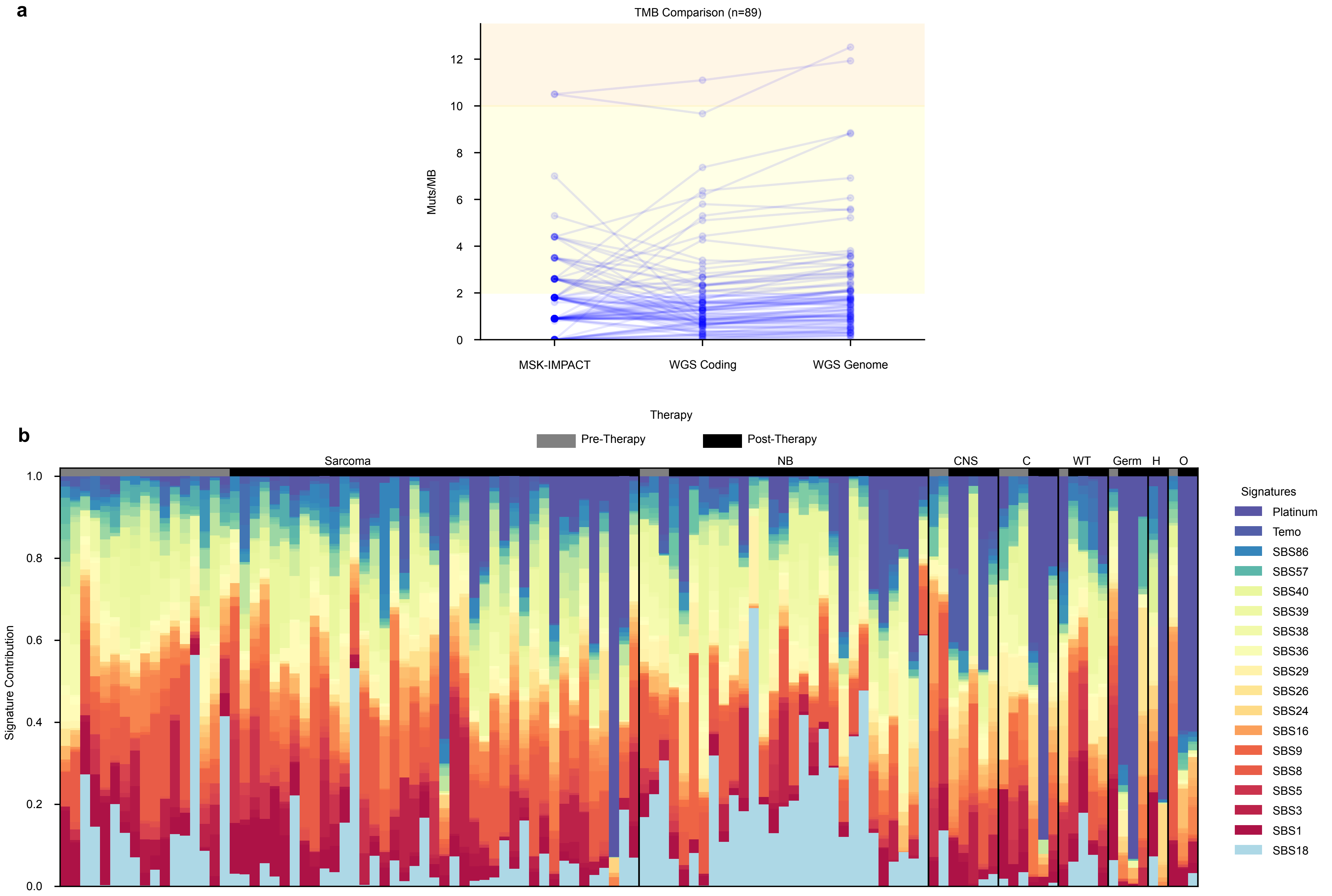
a
TMB Comparison (n=89)
Muts/MB
0
2
4
6
8
10
12
MSK-IMPACT
WGS Coding
WGS Genome
b
Therapy
Pre-Therapy
Post-Therapy
Sarcoma
NB
CNS
C
WT
Germ
H
O
Signature Contribution
0.0
0.2
0.4
0.6
0.8
1.0
Signatures
Platinum
Temo
SBS86
SBS57
SBS40
SBS39
SBS38
SBS36
SBS29
SBS26
SBS24
SBS16
SBS9
SBS8
SBS5
SBS3
SBS1
SBS18

Supplementary Figure 8: Genome-wide signals a) Tumor mutation burden (TMB) assessed by IMPACT (coding mutations), WGS (coding SNVs and Indels divided by exome), WGS genome wide (all SNVs and Indels divided by whole genome). b) Mutational signature contributions per sample. Signatures associated with Platinum and Temozolomide were merged respectively. Treatment status is annotated at the top. NB, neuroblastoma. CNS, central nervous system. C, carcinoma. WT, Wilms' tumor. Germ, germ cell tumor. H, hepatoblastoma. O, other. Source data for this figure are provided at the data repository.

S. Figure 9a

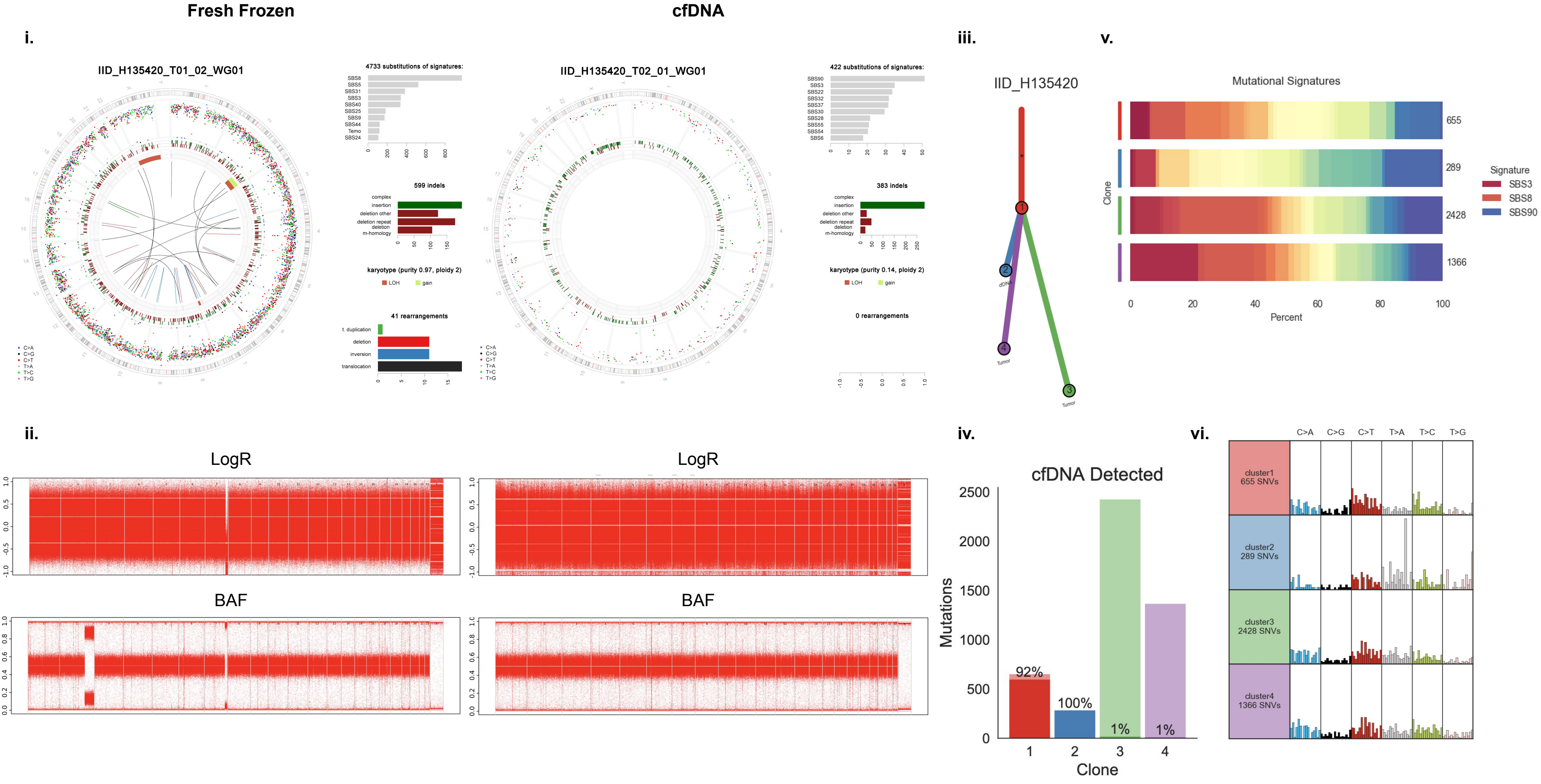
Fresh Frozen
cfDNA
i.
IID_H135420_T01_02_WG01
4733 substitutions of signatures:
599 indels
karyotype (purity 0.97, ploidy 2)
41 rearrangements
IID_H135420_T02_01_WG01
422 substitutions of signatures:
383 indels
karyotype (purity 0.14, ploidy 2)
0 rearrangements
iii.
IID_H135420
v.
Mutational Signatures
Clone
Percent
Signature
SBS3
SBS8
SBS90
ii.
LogR
BAF
iv.
cfDNA Detected
Mutations
Clone
92%
100%
1%
1%
vi.
cluster1
655 SNVs
cluster2
289 SNVs
cluster3
2428 SNVs
cluster4
1366 SNVs

S. Figure 9b

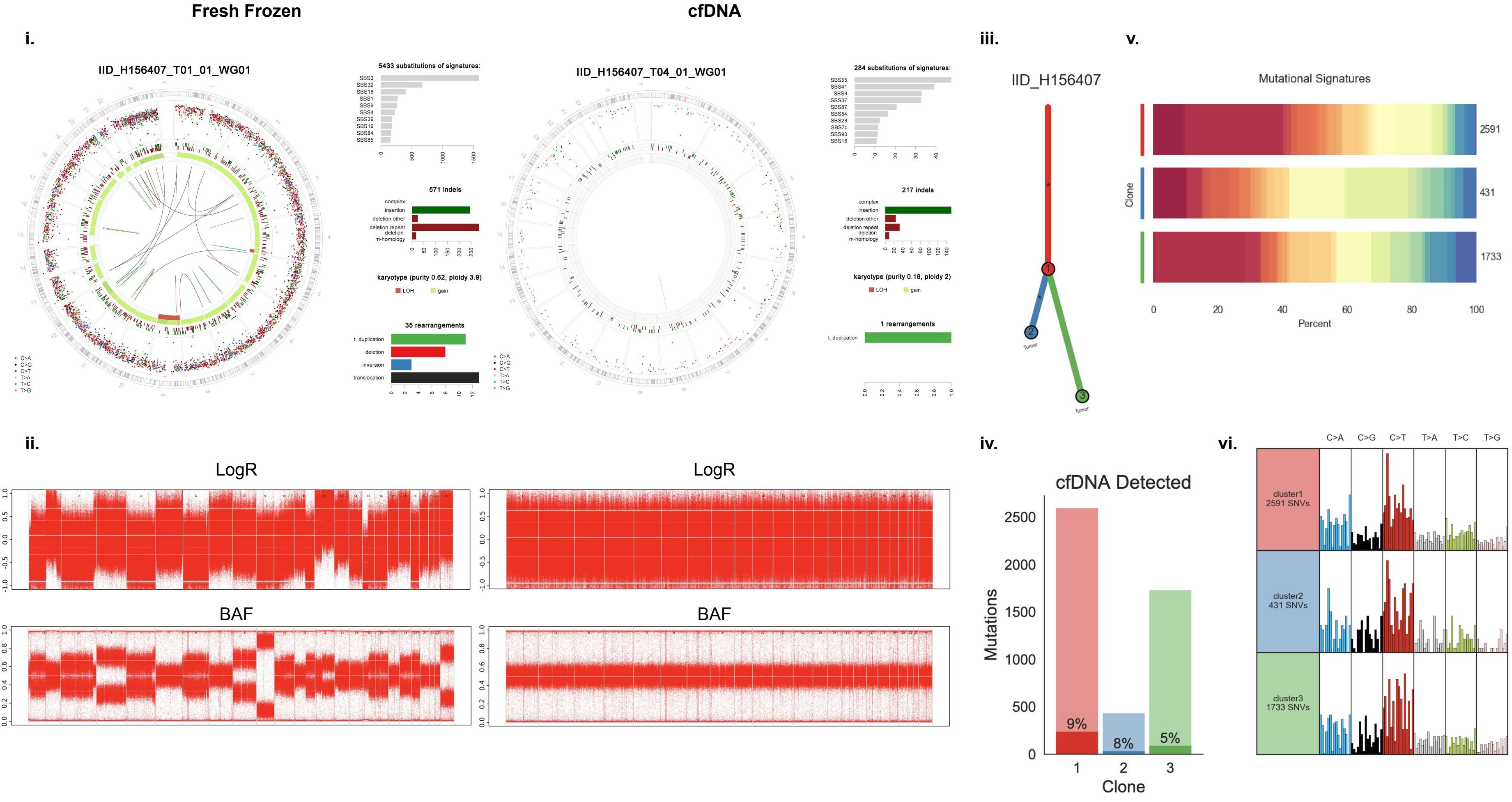
Fresh Frozen
cfDNA
i.
IID_H156407_T01_01_WG01
5433 substitutions of signatures:
571 indels
karyotype (purity 0.62, ploidy 3.9)
35 rearrangements
IID_H156407_T04_01_WG01
284 substitutions of signatures:
217 indels
karyotype (purity 0.18, ploidy 2)
1 rearrangements
iii.
IID_H156407
v.
Mutational Signatures
Signature
SBS3
SBS32
SBS39
Percent
Clone
ii.
LogR
BAF
iv.
cfDNA Detected
Mutations
Clone
vi.
cluster1 2591 SNVs
cluster2 431 SNVs
cluster3 1733 SNVs

S. Figure 9c

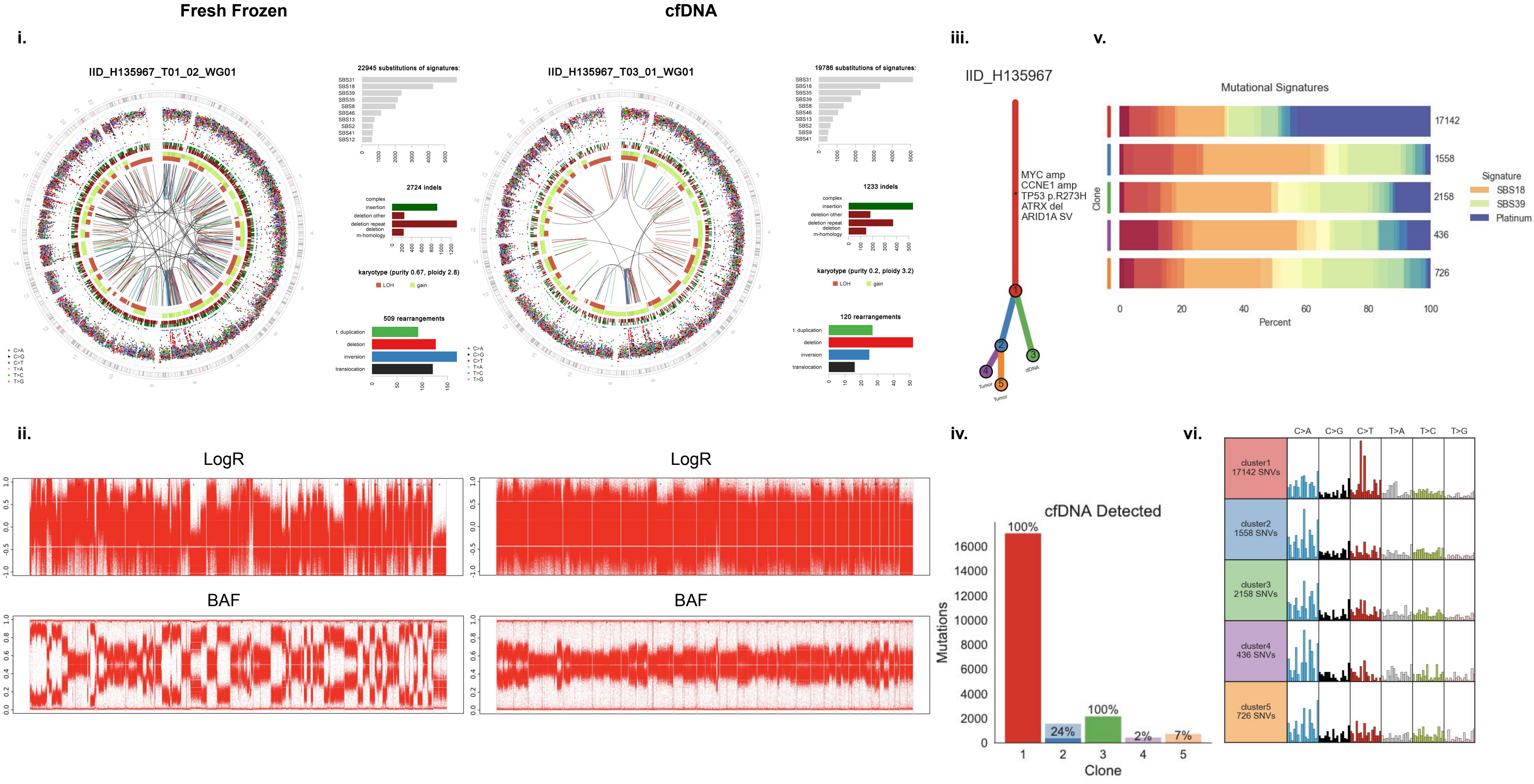
Fresh Frozen
cfDNA
i.
IID_H135967_T01_02_WG01
22945 substitutions of signatures:
SBS31
SBS18
SBS39
SBS35
SBS8
SBS46
SBS13
SBS2
SBS41
SBS12
2724 indels
complex
insertion
deletion other
deletion repeat
deletion
m-homology
karyotype (purity 0.67, ploidy 2.8)
LOH
gain
509 rearrangements
t. duplication
deletion
inversion
translocation
C>A
C>G
C>T
T>A
T>C
T>G
IID_H135967_T03_01_WG01
19786 substitutions of signatures:
SBS31
SBS18
SBS35
SBS39
SBS8
SBS46
SBS13
SBS2
SBS9
SBS41
1233 indels
karyotype (purity 0.2, ploidy 3.2)
120 rearrangements
iii.
IID_H135967
MYC amp
CCNE1 amp
TP53 p.R273H
ATRX del
ARID1A SV
Tumor
Tumor
cfDNA
v.
Mutational Signatures
Clone
17142
1558
2158
436
726
Percent
Signature
SBS18
SBS39
Platinum
ii.
LogR
LogR
BAF
BAF
iv.
cfDNA Detected
Mutations
100%
24%
100%
2%
7%
Clone
vi.
C>A
C>G
C>T
T>A
T>C
T>G
cluster1
17142 SNVs
cluster2
1558 SNVs
cluster3
2158 SNVs
cluster4
436 SNVs
cluster5
726 SNVs

S. Figure 9d

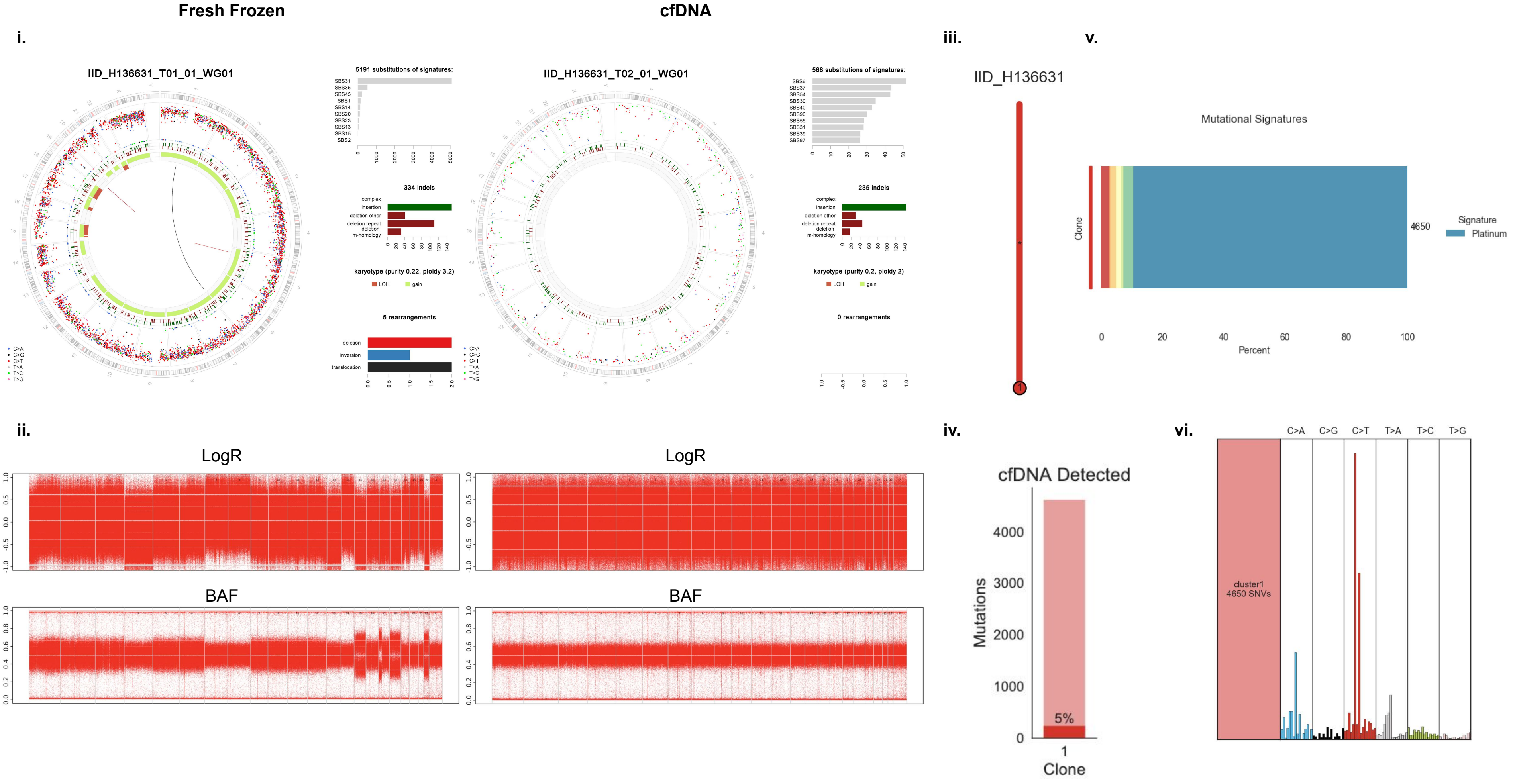
Fresh Frozen
cfDNA
i.
IID_H136631_T01_01_WG01
5191 substitutions of signatures:
334 indels
karyotype (purity 0.22, ploidy 3.2)
LOH
gain
5 rearrangements
deletion
inversion
translocation
IID_H136631_T02_01_WG01
568 substitutions of signatures:
235 indels
karyotype (purity 0.2, ploidy 2)
0 rearrangements
iii.
IID_H136631
v.
Mutational Signatures
Clone
4650
Signature
Platinum
Percent
ii.
LogR
BAF
iv.
cfDNA Detected
Mutations
5%
Clone
vi.
C>A
C>G
C>T
T>A
T>C
T>G
cluster1
4650 SNVs

S. Figure 9e

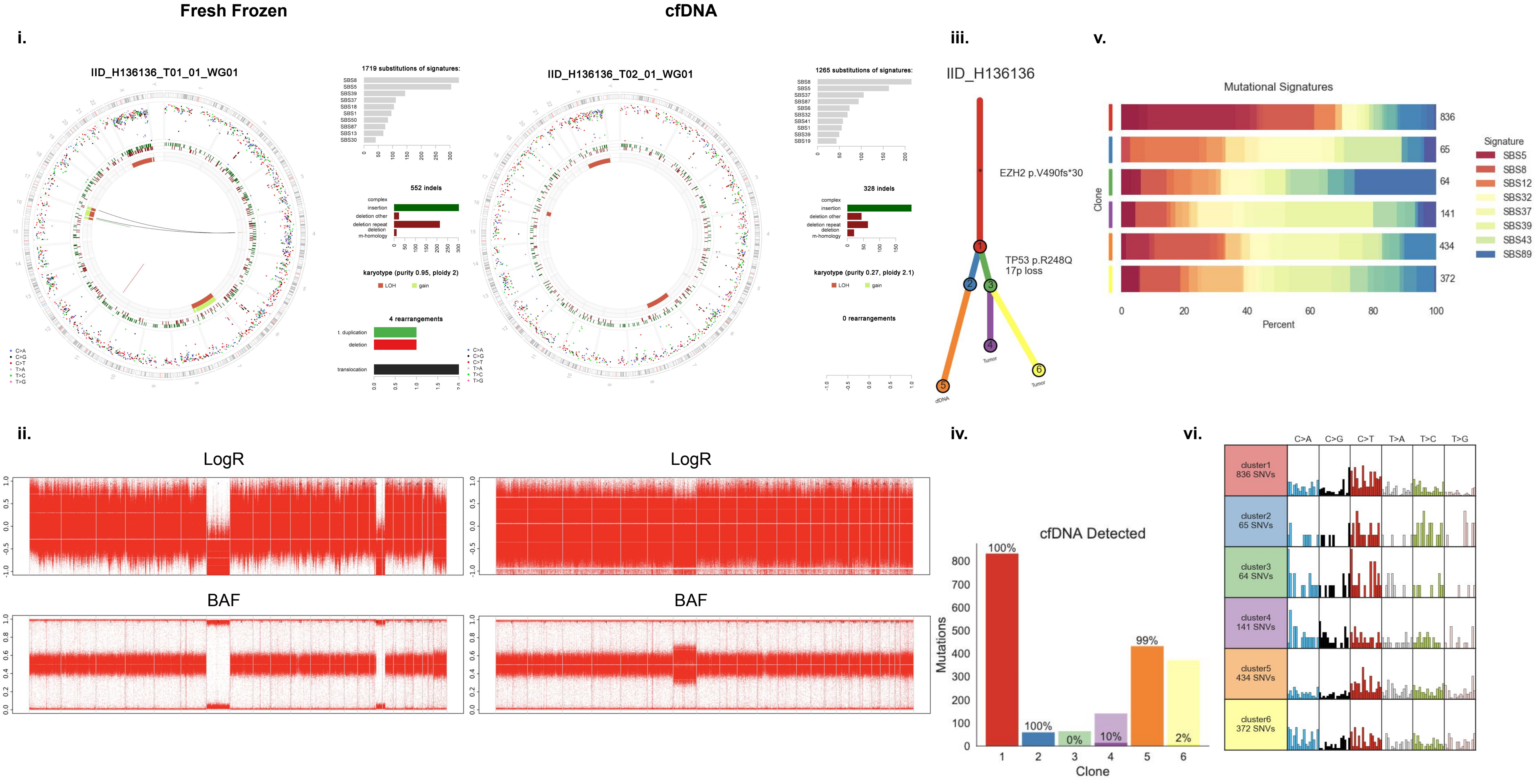

Fresh Frozen
cfDNA
i.
IID_H136136_T01_01_WG01
1719 substitutions of signatures:
SBS8
SBS5
SBS39
SBS37
SBS18
SBS1
SBS50
SBS87
SBS13
SBS30
552 indels
complex
insertion
deletion other
deletion repeat
deletion m-homology
karyotype (purity 0.95, ploidy 2)
LOH
gain
4 rearrangements
t. duplication
deletion
translocation
C>A
C>G
C>T
T>A
T>C
T>G
IID_H136136_T02_01_WG01
1265 substitutions of signatures:
SBS8
SBS5
SBS37
SBS87
SBS6
SBS32
SBS41
SBS1
SBS39
SBS19
328 indels
karyotype (purity 0.27, ploidy 2.1)
0 rearrangements
iii.
IID_H136136
EZH2 p.V490fs*30
TP53 p.R248Q
17p loss
Tumor
cfDNA
v.
Mutational Signatures
Clone
836
65
64
141
434
372
Percent
Signature
SBS5
SBS8
SBS12
SBS32
SBS37
SBS39
SBS43
SBS89
ii.
LogR
BAF
iv.
cfDNA Detected
100%
100%
0%
10%
99%
2%
Mutations
Clone
vi.
C>A
C>G
C>T
T>A
T>C
T>G
cluster1
836 SNVs
cluster2
65 SNVs
cluster3
64 SNVs
cluster4
141 SNVs
cluster5
434 SNVs
cluster6
372 SNVs

S. Figure 9f

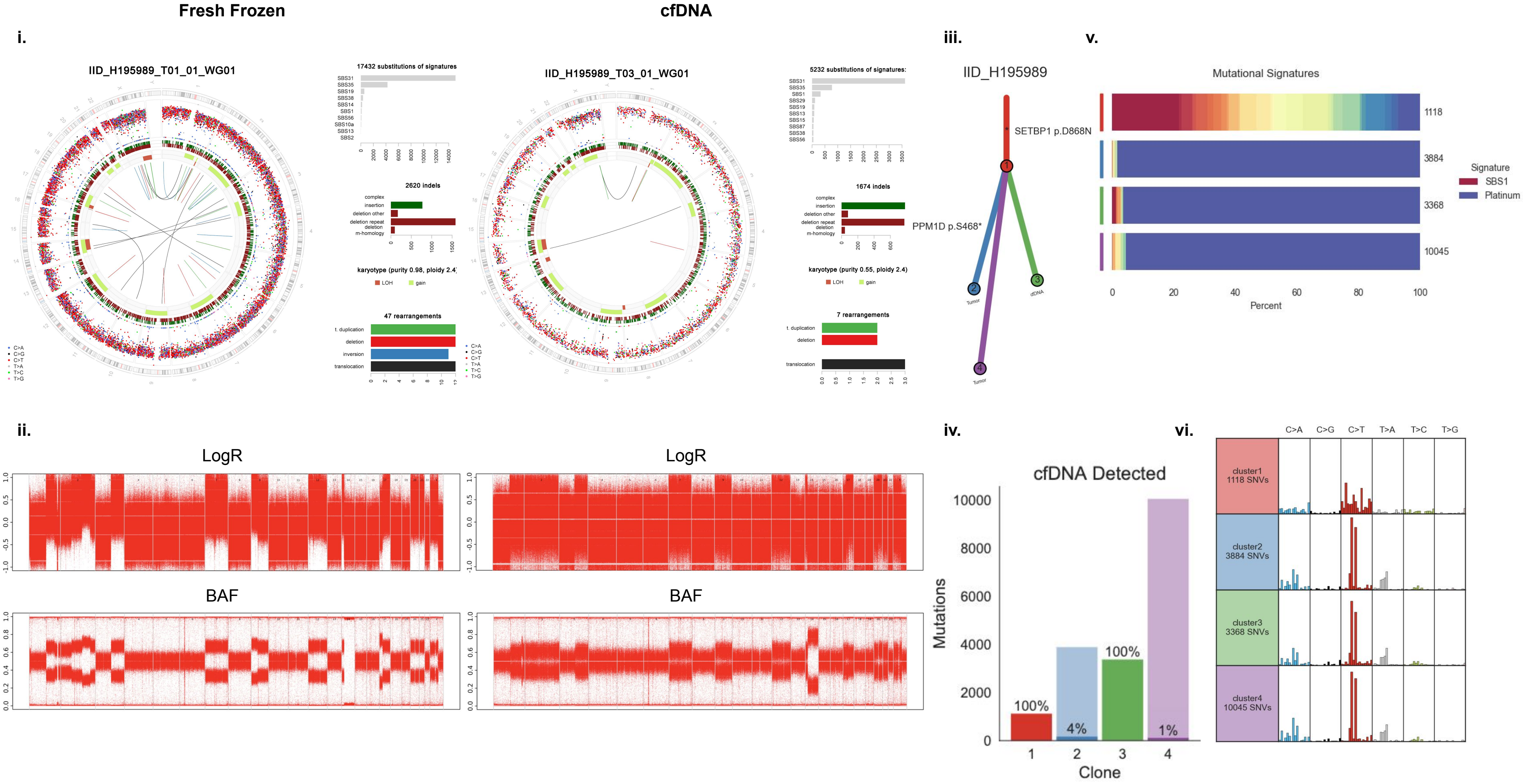
Fresh Frozen
cfDNA
i.
IID_H195989_T01_01_WG01
17432 substitutions of signatures
2620 indels
karyotype (purity 0.98, ploidy 2.4)
47 rearrangements
t. duplication
deletion
inversion
translocation
IID_H195989_T03_01_WG01
5232 substitutions of signatures:
1674 indels
karyotype (purity 0.55, ploidy 2.4)
7 rearrangements
ii.
LogR
BAF
iii.
IID_H195989
SETBP1 p.D868N
PPM1D p.S468*
iv.
cfDNA Detected
Mutations
Clone
100%
4%
100%
1%
v.
Mutational Signatures
1118
3884
3368
10045
Percent
Signature
SBS1
Platinum
vi.
C>A
C>G
C>T
T>A
T>C
T>G
cluster1
1118 SNVs
cluster2
3884 SNVs
cluster3
3368 SNVs
cluster4
10045 SNVs

S. Figure 9g

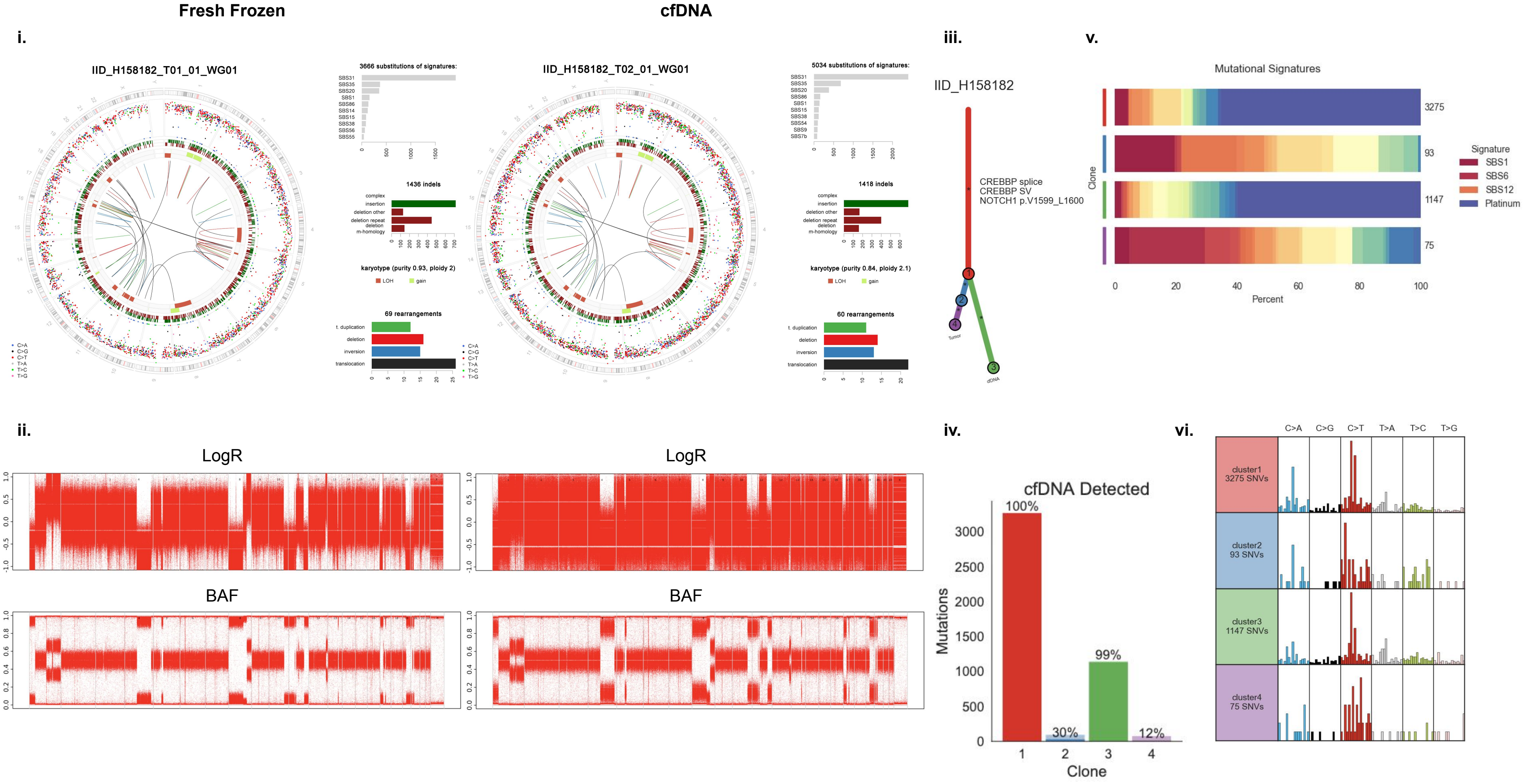
Fresh Frozen
cfDNA
i.
IID_H158182_T01_01_WG01
3666 substitutions of signatures:
1436 indels
karyotype (purity 0.93, ploidy 2)
69 rearrangements
IID_H158182_T02_01_WG01
5034 substitutions of signatures:
1418 indels
karyotype (purity 0.84, ploidy 2.1)
60 rearrangements
iii.
IID_H158182
CREBBP splice
CREBBP SV
NOTCH1 p.V1599_L1600
v.
Mutational Signatures
Signature
SBS1
SBS6
SBS12
Platinum
Clone
Percent
ii.
LogR
BAF
iv.
cfDNA Detected
Mutations
Clone
100%
30%
99%
12%
vi.
cluster1 3275 SNVs
cluster2 93 SNVs
cluster3 1147 SNVs
cluster4 75 SNVs
C>A
C>G
C>T
T>A
T>C
T>G

Supplementary Figure 9: cfDNA analysis extended a-g) Individual level summary comparisons for matched cfDNA and tissue samples in order of purity where i) shows Circos plots as described in Figure 5, ii) LogR and BAF tumor profiles from cgpBattenberg, iii) individual level phylogenetic tree or tissue phylogenetic tree if no cfDNA subclones (H156407, H136631) with clinically relevant variants annotated, iv) bar plot of clusters estimated by substitutions with percentage of variants with pileup support from cfDNA shaded and annotated, v) mutational signature exposures per same clusters, vi) 96 mutation contexts for each cluster. Raw data for this figure can be accessed at the dbGAP study.
