# Supplementary material for: Feasibility of whole genome and transcriptome profiling in pediatric and young adult cancers
Source: Nat Commun. 2022 May 18;13:2485. doi: 10.1038/s41467-022-30233-7 (PMC9117241; doi:10.1038/s41467-022-30233-7)
Supplement: Supplementary file 3 — Description of Additional Supplementary Information [file 41467_2022_30233_MOESM3_ESM.pdf]

Supplementary Data Legends

Supplementary Data 1. Study cohort characteristics.

Supplementary Data 2. Quality control metrics for WGS and RNAseq data for the study cohort. The column named "DNA GC dropout" refers to GC_GROPOUT as calculated by Picard.

Supplementary Data 3. Totality of findings by cWGTS with annotation on clinical relevance. For column 'Any previous clinical finding', 0: No prior finding by clinical diagnostic workflow and no additional by cWGTS, 1: No prior finding by clinical diagnostic workflow and additional by cWGTS, 2: Prior finding by clinical diagnostic workflow, no additional finding by cWGTS, 3: Prior finding by clinical diagnostic workflow and additional finding by cWGTS. SV, structural variant. TMB, tumor mutation burden. MSI, microsatellite instability. WGD, whole genome duplication. Telomere, telomere lengthening or shortening.

Supplementary Data 4. Sequencing metrics for mutations (substitutions and indels) reported by MSK-IMPACT and corresponding data in WGS sequencing and validation assays. VAF, variant allele frequency. CCF, cancer cell fraction. ITH, intratumor heterogeneity.

Supplementary Data 5. Summary of germline mutations identified in cohort

Supplementary Data 6. Fusion events identified by MSK-IMPACT/MSK-FUSION/cWGTS. A value of 0 indicates not assessed, 1: Not called, 2:Supporting evidence and * please see supporting note.

Supplementary Data 7. Median coverages for each downsampled sample at each level

Supplementary Data 8. Somatic SNV/Indel driver mutations in cancer genes identified only by WGS. VAF, variant allele frequency. CCF, cancer cell fraction. Mutation copy number, number of chromosome bearing mutation.

Supplementary Data 9. Somatic Structural Variant (SV) driver mutations identified by WGS. TRA, translocation. DEL, deletion. DUP, duplication. INV, inversion.

Supplementary Data 10. Expression biomarkers identified by RNA using methodology from Horak et al. 2021.

Supplementary Data 11. Summary findings of matched cfDNA and fresh frozen (FF) specimens. For Genome, a value of 2 represents full recapitulation, 1: some recapitulation, 0: no recapitulation.
