# Supplementary material for: Feasibility of whole genome and transcriptome profiling in pediatric and young adult cancers
Source: Nat Commun. 2022 May 18;13:2485. doi: 10.1038/s41467-022-30233-7 (PMC9117241; doi:10.1038/s41467-022-30233-7)
Supplement: Supplementary file 15 — Reporting Summary [file 41467_2022_30233_MOESM15_ESM.pdf]

nature portfolio

Corresponding author(s): Elli Papaemmanuil

Last updated by author(s): Mar 30, 2022

Reporting Summary

Nature Portfolio wishes to improve the reproducibility of the work that we publish. This form provides structure for consistency and transparency in reporting. For further information on Nature Portfolio policies, see our Editorial Policies and the Editorial Policy Checklist.

Statistics

For all statistical analyses, confirm that the following items are present in the figure legend, table legend, main text, or Methods section.

n/a	Confirmed	
☐	☒	The exact sample size (*n*) for each experimental group/condition, given as a discrete number and unit of measurement
☒	☐	A statement on whether measurements were taken from distinct samples or whether the same sample was measured repeatedly
☐	☒	The statistical test(s) used AND whether they are one- or two-sided<br>*Only common tests should be described solely by name; describe more complex techniques in the Methods section.*
☒	☐	A description of all covariates tested
☒	☐	A description of any assumptions or corrections, such as tests of normality and adjustment for multiple comparisons
☐	☒	A full description of the statistical parameters including central tendency (e.g. means) or other basic estimates (e.g. regression coefficient) AND variation (e.g. standard deviation) or associated estimates of uncertainty (e.g. confidence intervals)
☒	☐	For null hypothesis testing, the test statistic (e.g. *F*, *t*, *r*) with confidence intervals, effect sizes, degrees of freedom and *P* value noted<br>*Give P values as exact values whenever suitable.*
☒	☐	For Bayesian analysis, information on the choice of priors and Markov chain Monte Carlo settings
☒	☐	For hierarchical and complex designs, identification of the appropriate level for tests and full reporting of outcomes
☐	☒	Estimates of effect sizes (e.g. Cohen's *d*, Pearson's *r*), indicating how they were calculated

Our web collection on statistics for biologists contains articles on many of the points above.

Software and code

Policy information about availability of computer code

Data collection	No code was used to collect data
Data analysis	Scripts for generating the figures are provided at https://github.com/papaemmelab/Shukla_Levine_Gundem

For manuscripts utilizing custom algorithms or software that are central to the research but not yet described in published literature, software must be made available to editors and reviewers. We strongly encourage code deposition in a community repository (e.g. GitHub). See the Nature Portfolio guidelines for submitting code & software for further information.

Data

Policy information about availability of data

All manuscripts must include a data availability statement. This statement should provide the following information, where applicable:

- Accession codes, unique identifiers, or web links for publicly available datasets
- A description of any restrictions on data availability
- For clinical datasets or third party data, please ensure that the statement adheres to our policy

The raw data for WGS and RNA-seq data generated in this study have been deposited in the dbGAP database under accession code phs002620.v1.p [http://www.ncbi.nlm.nih.gov/projects/gap/cgi-bin/study.cgi?study_id=phs002620.v1.p1]. These data are available under restricted access due individual privacy concerns. Permanent employees of an institution at a level equivalent to a tenure-track professor or senior scientist with laboratory administration and oversight responsibilities may request access through dbGAP. The requests, which are managed by NCI's Data Access Committee, take less than 2 days for approval and access is permitted for 12-months. The processed MSK-IMPACT data are available in a study-specific dataset at cbioPortal [https://www.cbioportal.org/study/summary?id=mixed_kunga_msk_2022]. Summary and processed data for the figures are available in the source data file as well as the data repository at https://

github.com/papaemmelab/Shukla_Levine_Gundem. Annotation databases included public resources such as Cancer Gene Census, OncoKb, ClinVar, 1000genomes, gnomAD, and Ensembl Variant Effect Predictor (VEP) databases. The remaining data are available within the Article and Supplementary Information file.

Field-specific reporting

Please select the one below that is the best fit for your research. If you are not sure, read the appropriate sections before making your selection.

☒ Life sciences ☐ Behavioural & social sciences ☐ Ecological, evolutionary & environmental sciences

For a reference copy of the document with all sections, see nature.com/documents/nr-reporting-summary-flat.pdf

Life sciences study design

All studies must disclose on these points even when the disclosure is negative.

Sample size	No statistical method was performed to determine sample size.
Data exclusions	All data was included in the analyses.
Replication	No experiment was performed.
Randomization	No randomization was performed.
Blinding	This is not applicable for our study.

Reporting for specific materials, systems and methods

We require information from authors about some types of materials, experimental systems and methods used in many studies. Here, indicate whether each material, system or method listed is relevant to your study. If you are not sure if a list item applies to your research, read the appropriate section before selecting a response.

Materials & experimental systems

n/a	Involved in the study	
☒	☐	Antibodies
☒	☐	Eukaryotic cell lines
☒	☐	Palaeontology and archaeology
☒	☐	Animals and other organisms
☐	☒	Human research participants
☐	☒	Clinical data
☒	☐	Dual use research of concern

Methods

n/a	Involved in the study	
☒	☐	ChIP-seq
☒	☐	Flow cytometry
☒	☐	MRI-based neuroimaging

Human research participants

Policy information about studies involving human research participants

Population characteristics	Patients who were seen within the Department of Pediatrics at Memorial Sloan Kettering Cancer Center with presumed or established solid tumor malignancies (including CNS tumors).
Recruitment	Patients were enrolled on an institutional prospective tumor/germline sequencing protocol (ClinicalTrials.gov number, NCT01775072) with informed consent from patients or their guardians.
Ethics oversight	Ethics oversight was performed by the institutional review board at MSKCC.

Note that full information on the approval of the study protocol must also be provided in the manuscript.

Clinical data

Policy information about clinical studies

All manuscripts should comply with the ICMJE guidelines for publication of clinical research and a completed CONSORT checklist must be included with all submissions.

Clinical trial registration	NCT01775072
Study protocol	Not applicable.

Data collection	Not applicable.
Outcomes	Not applicable.
